# Beyond Sight: The Influence of Opaque Glasses on Wine Sensory Perception

**DOI:** 10.3390/foods14183231

**Published:** 2025-09-17

**Authors:** George Ștefan Coman, Camelia Elena Luchian, Elena Cristina Scutarașu, Valeriu V. Cotea

**Affiliations:** “Ion Ionescu de la Brad” Iasi University of Life Sciences, 3rd M. Sadoveanu Alley, 700490 Iași, Romania; george.coman@iuls.ro (G.Ș.C.); cristina.scutarasu@iuls.ro (E.C.S.); valeriu.cotea@iuls.ro (V.V.C.)

**Keywords:** blind tasting, organoleptic study, food choice, consumer perception, maceration

## Abstract

International standards for wines with Protected Designation of Origin (PDO) require characterisation through both analytical and sensory criteria, although sensory evaluation remains inherently subjective, especially regarding organoleptic properties. This study examined paired Blanc de noir and red wines made from identical grape varieties to determine whether varietal traits remain perceptible regardless of the vinification method while also assessing the role of visual stimuli in influencing olfactory and gustatory perception. Controlled tastings were conducted using both transparent and opaque glassware, with experienced panellists recording sensory descriptors. Physicochemical parameters were measured using a Lyza 5000 analyser to confirm compliance with quality standards, while statistical analyses of sensory data were conducted using the XLSTAT–Basic, student-type user software. Results showed that the absence of visual cues did not mislead tasters in recognising core attributes; however, the winemaking method significantly affected descriptors linked to maceration, including flavour intensity, astringency, and red/dark fruit notes. Panellists distinguished between white and red wines at statistically significant levels, even without visual input, suggesting that vinification-related chemical composition primarily guided their perception. Direct correlations were observed between red winemaking descriptors and parameters such as pH, lactic acid, glycerol, and volatile acidity, while indirect correlations were found with malic acid and titratable acidity. The results highlight how winemaking methods, chemical composition, and sensory perception interact in defining varietal characteristics.

## 1. Introduction

A search for published studies on topics such as ‘sensory differences’, ‘white wine’, ‘red wine’, and ‘tasting bias’ revealed that only two studies had been published [1]. Among the earliest studies on olfactory perceptions related to wine’s chromatic properties were those by Ballester and colleagues at the Université de Bourgogne [2]. In 2009, these researchers conducted sensory evaluations of eighteen premium wines (six white, six rosé, and six red) from French viticultural regions using the 2015 harvest of various grape varieties obtained through commercial retail channels. The main findings indicated that, regardless of evaluator expertise, the olfactory profiles of white and red wines were accurately identified, irrespective of the presence of visual stimuli. Conversely, rosé wines posed significant identification challenges [2].

Subsequent investigations within this limited research domain have established that visual parameters, particularly chromatic characteristics, constitute critical determinants in olfactory and gustatory perception of alimentary products and beverages, as initial sensory impressions form during primary exposure [3]. The previous conclusion also supports the fact that humans can accurately recognise approximately a third of familiar orthonasal odours when these cues are presented alone [4]. Cross-modal sensory interactions exemplify this phenomenon, particularly the association between olfactory and gustatory attributes, wherein numerous distinct aromatic characteristics receive qualitative gustatory descriptors [5]. Furthermore, individuals experiencing temporary anosmia frequently report concurrent partial or complete dysgeusia [6], underscoring the interconnected nature of chemosensory modalities in perceptual processing.

Predicting the overall odour profile of wine only from its chemical composition is a highly complex and arguably unachievable task, as sensory perception arises from more than just the additive effects of individual compounds [7]. The interaction between volatile and non-volatile constituents is significant because specific molecules can enhance, mask, or suppress others depending on their relative concentrations and interactions. External factors, such as temperature, also influence volatility, release rates, and perceptual thresholds, thereby affecting the aromatic expression of the compound. Furthermore, matrix effects—caused by the presence of ethanol, sugars, and macromolecules—can significantly impact the availability, partitioning, and delivery of volatile compounds to olfactory receptors. Collectively, these interactions emphasise the multifactorial and dynamic nature of odour perception in wine, where chemical composition, molecular interactions, and environmental conditions all contribute to shaping the final sensory experience.

Over one thousand organic and inorganic components, identified as present in wine, continuously interact with each other in pursuit of a perpetual dynamic equilibrium [8]. This entire ‘storm’ of interconnected compounds, grouped into several main categories, generally follows this structure: water/86%, ethanol/12%, glycerol/4–10 g/L, higher alcohols/300–600 mg/L, polysaccharides/1%, organic acids/0.4%, polyphenols/1–5 g/L in red wines or 0.2–0.5 g/L in white wines [9], minerals/1.5–3 g/L, volatile compounds and other compounds—0.5% [10], and, in effect, creates a sensory flux that is perceived differently during organoleptic evaluation, depending on the tasting context and the sensory memory of each taster.

Research published by Gottfried in 2003 has shown that when visual and olfactory stimuli do not align, the odour attributes of wine have limited influence on discrimination. Notably, studies conducted at the University of Bordeaux demonstrated that visual cues can even override olfactory perception. When a wine made through ‘white winemaking’ was coloured with an odourless red dye, it was mainly described using descriptors typical of red wines, especially by tasters with some oenological training [11].

While consensus models remain central to wine sensory analysis, emerging evidence suggests that individual variability among tasters—shaped by physiology, prior experience, and knowledge—plays a fundamental role in sensory evaluation [12]. Emotionally salient odours, such as those associated with early life experiences, are deeply encoded in sensory memory and strongly influence perceptual and hedonic responses [13]. Consequently, the practice of training tasters to apply standardised descriptors may enhance linguistic uniformity but does not guarantee conceptual consistency, as perceptual and cognitive associations with specific odours remain highly individual [14].

A key factor with significant implications for wine sensory evaluation is the conclusion of a 2014 study, conducted by Köster, Møller, and Mojet, which shows that olfactory–induced influences (specifically, behavioural changes triggered by exposure to odours) can affect individuals even when they are not consciously aware of the odour’s presence or its impact on their behaviour. This suggests that our sensory and cognitive responses to wine are shaped not only by consciously perceived aromas but also by implicit olfactory cues that operate beyond our awareness. Consequently, individuals may be unable to explicitly recognise, articulate, or even identify the specific odour-related influences underlying their perception, appreciation, and judgment of a wine. These findings challenge traditional assumptions about sensory objectivity in wine evaluation and emphasise the complexity of olfactory perception in shaping subjective experiences [15].

After analysing research from Université de Bourgogne [2], which examined various white, rosé, and red wines, the question arises: what would happen if wines were produced from the same black grape variety? Refining all previously published research findings consulted during the development of the working hypotheses for this study, the main aim was to identify how sensory perceptions of wines are affected by the absence of the visual chromatic cue and, additionally, by different winemaking methods applied to the same grape variety. One hypothesis was that the specific types of different winemaking methods are reflected in physicochemical differences inherent to the wine’s nature, enough to be perceptible by the senses even when the wines are tasted from opaque glasses. Given the Regulation (EU) No 1308/2013 of the European Parliament and of the Council of 17 December 2013 [16], which mandates the sensory description of wines registered as ‘Protected Designation of Origin’, it also seeks to investigate the hypothesis that there are sensory elements inherent to wine itself that are independent of early life or prior experience of tasters. Ultimately, it was meant to verify the hypothesis that sensory traits usually associated with red wines can be identified in white wines made from the same black grape variety when visual cues are absent.

## 2. Materials and Methods

### 2.1. Wine Samples

Thirteen commercial wine samples were purchased online from authorised sellers over several months during the first semester of 2023. Efforts were made to minimise variability unrelated to the winemaking process, both in the physicochemical parameters and sensory perception of the wines, by selecting a ‘flight’ in which the wines were vinified as similarly as possible, originating from the same grape varieties; sourced from the same winery; and harvested in the same year. Each wine sample was provided with three bottles. The bottles had been checked beforehand to ensure they were free from faults. Wines produced in two styles were selected: ‘Blanc de noir’, made using white winemaking methods from black-skinned grapes, and ‘Red’, characterised mainly by features developed during the maceration stage of red winemaking. One of the samples was a ‘Rosé’ wine, made from the same black grapes used for ‘Blanc de noir’ and red wines, through direct pressing. Most of the wine samples were monovarietal, except for those with the conventional IDs V12 and V13, which were produced by blending 95% Touriga Franca and 5% Syrah grapes, as shown in Table 1. ID represents a conventional number assigned to each sample, remaining consistent across all tasting sessions and not indicating the order of serving for the tasters’ analysis. In the Region column, the first two letters denote the country-of-origin code of the winery (and implicitly the wine) in Alpha-2 format, followed by the name of the DOP region, which encompasses all wines of that quality level.

Beyond simply identifying samples by labels, the wine samples were analysed from a physicochemical perspective using a multi–parameter wine analyser, Lyza 5000 (Anton Paar, Graz, Austria) [17]. Infrared spectroscopy offers an economical method for analysing grapes and wines. Originally, the purpose of infrared spectroscopy in relation to wines was to determine alcohol and sugar content [18]. In this study, the common physicochemical parameters of wines were analysed using Fourier transform infrared (FTIR) spectroscopy with attenuated total reflectance (ATR). ATR is based on the principle of total internal reflection, where infrared (IR) radiation interacts with the sample only at the interface where reflection occurs. In contrast, transmission spectroscopy depends on IR radiation passing directly through the sample [19]. The parameters assessed—alcohol content (% *v*/*v*), total glucose and fructose (g/L), titratable acidity (g/L, expressed as tartaric acid equivalents), volatile acidity (g/L, expressed as acetic acid), malic acid (g/L), tartaric acid (g/L), lactic acid (g/L), pH, density (g/mL), total dry extract (g/L), glycerol (g/L), and total polyphenols (mg/L)—were determined automatically by the analytical equipment in less than two minutes per sample [20]. This was achieved through a single ampoule injection, in accordance with the protocol established in the Compendium of International Methods of Wine and Must Analysis [21]. The rapidity, accuracy, and reproducibility of this method have contributed to its validation as a dependable tool for the comprehensive analysis of wine and must [22].

### 2.2. Tasters Panel

The size of the panel depends on the experimental design and assessor competence. According to the OIV Standard for International Wine and Spirituous Beverages of Vitivinicultural Origin Competitions, Ed. 2021, states in Article 7—Designation of Jurors that ‘In principle, each jury shall be composed of 7 jurors.’ In sensory evaluation literature, a panel of 15–20 trained tasters is generally recommended, as this guarantees at least twelve participants per session. Conversely, panels smaller than five tend to produce unreliable statistical results [23]. Twenty-one qualified tasters were selected, of whom eleven were female and willing to participate in the study. The term ‘qualified’ was used based on their involvement in the wine industry, where they regularly assess wines; their professional duties included teaching university courses in oenology and viticulture, conducting scientific research in the beverage sector, holding O.I.V. expert status, accreditation as Romanian authorised tasters or sommeliers, or possessing at least a Wine & Spirit Education Trust Level 2 certification [24]. Some of them graduated from the master’s programme in beverages at Iasi University of Life Sciences. The tasters were informed beforehand that they would be sampling alcoholic beverages, and all were of legal drinking age at the time of the tasting sessions. All participants adhered to an ethical informed consent process in accordance with the ethical code of Iasi University of Life Sciences.

### 2.3. Design of the Experiment

**Figure 1:**
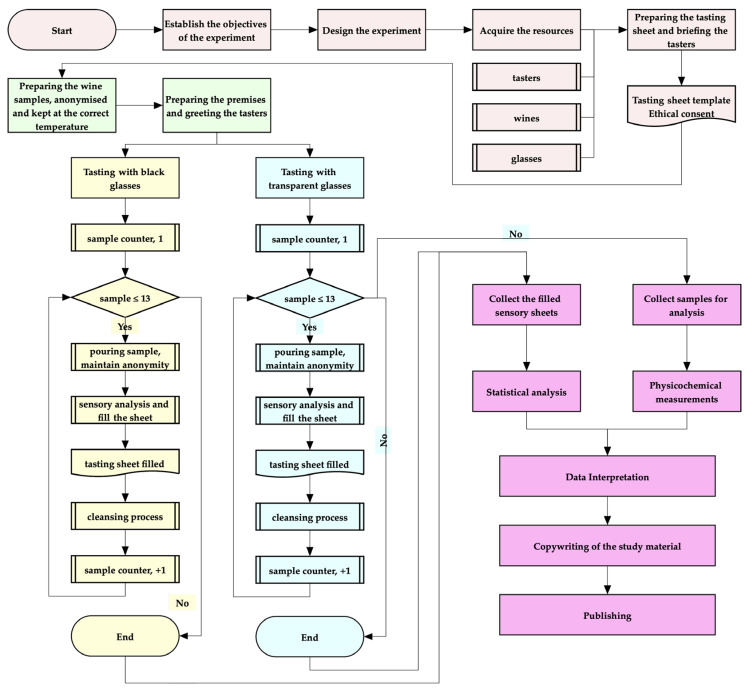
Graphical representation of the experimental design.

To ensure the proper progression of the study and to maintain a consistent focus on the objectives, a flowchart was created. The experiment’s processes are illustrated in Figure 1.

After establishing the main parameters of the experiments, the next step was to gather the necessary resources. The design of the tasting sheet was based on the Wine & Spirits Education Trust (Level 4) Systematic Approach [25], modified to include specific features that reflect the quantified intensity of the maceration stage. To achieve an optimal synthesis (both representative and statistically meaningful) of the sensory data collected by the tasters, the following organoleptic characteristics were selected and refined, as shown in Table 2.

The design of the tastings was arranged to minimise the risk of arbitrary influence on the research outcomes [27] and aimed to ensure complete anonymisation of the tasters by assigning random IDs. This approach was intended to prevent the perception that the tasting sessions were tests of the tasters’ knowledge or the panel members’ ability to interpret or recall instructions. When critically evaluating wines, withholding information such as varietal, stylistic, or regional origin, price, or producer is essential to avoid bias in assessments, whether positive or negative [23]. The sensory analysis followed the OIV recommendations [28]. The tasting sessions were conducted under conditions where tasters were unaware of the wines’ origins or other identifying features. Before each session, all bottles were fully anonymised and randomly mixed; only one person, not among the tasters, who poured into the glasses, knew the true identity of each wine sample. Regarding the serving sequence, the white and red wine samples were combined. This method was used to prevent guesswork about the type of wine and to minimise sensory bias, which could be influenced solely by the sample’s general aspects category. Additionally, the preferred order, within feasible limits, followed the increasing levels of alcohol concentration and ageing period. Therefore, the chosen sequence was: (1) Rosé (13% ABV, 2022), (2) White (12% ABV, 2021), (3) White (13% ABV, 2021), (4) Red (13% ABV, 2021), (5) Red (11.5% ABV, 2019), (6) Red (14.5% ABV, 2020), (7) White (12.5% ABV, 2021), (8) White (12% ABV, 2019), (9) White (13% ABV, 2021), (10) White (12.5% ABV, 2021), (11) Red (14% ABV, 2017), (12) Red (14.5% ABV, 2019), (13) Red (14% ABV, 2020).

During the initial tasting sessions, opaque glasses were used to prevent any visual bias, and the lighting was kept at a minimum to enable accurate sensory notes to be recorded on the tasting sheet. Tasters were unaware of the bottle shapes. Red napkins concealed the colour of the drink, while plain water, slices of natural bread, and Granny Smith apple pieces served as palate cleansers. The tasting sessions began at 10 a.m. and lasted approximately two hours. At the end of each session, the true identity of the samples was not disclosed to maintain the impartiality of the tasters and preserve the integrity of the tasting environment. Given the sample numbers and the multiple sensory attributes to evaluate, a five–point Likert scale was employed to measure the intensity of sensory perceptions. This choice was made because the scale is accessible and efficient for data collection. The tool facilitates the collection of quantitative data suitable for statistical analysis, although responses may be affected by biases such as central tendency error or extreme response style, which could influence participant evaluations. 

### 2.4. Data Analysis

To analyse the data statistically, XLSTAT–Basic XLSTAT–Basic, student-type user software, a statistical and data analysis solution (Lumivero, Denver, CO, USA), was used. Since fewer than 50 unique experimental data observations were collected, deviations from a normal distribution could significantly impact the results, primarily leading to false positives, as the Central Limit Theorem, which states that the distribution of the mean tends towards normality regardless of the original distribution, cannot be applied. Therefore, significance testing of the ‘*p*-value’ for the wines as a source of variance was first performed using a Shapiro–Wilk test. The ‘W’ statistic from Shapiro–Wilk indicates that the closer it is to one, the more the data resemble a normal distribution. The ‘*p*-value’ represents the probability of obtaining such data under the null hypothesis (H0), which states that the residuals are normally distributed. The higher its value is than a conventional significance threshold (α = 0.05), the less evidence there is to reject the null hypothesis. Subsequently, a univariate analysis of variance (ANOVA) was carried out for each attribute using a one–way model, with the descriptors as fixed variables and the wine as a random effect. The descriptors were analysed separately in the two different tasting contexts, using opaque and transparent glasses. To examine the relationships between sensorial descriptors, Kendall correlation tests were conducted on attribute values scored by tasters, specifically during tasting sessions with opaque glasses and those with transparent glasses. Kendall’s correlation was deemed the most suitable method for analysing the relationships between sensory descriptors because of the limited amount of unique experimental data collected. Visual representations of the wines in various tasting contexts were generated using Principal Component Analysis (PCA) applied to the covariance matrices of the mean ratings for descriptor intensities, calculated across the entire panel of tasters for each wine. The selection of principal components was primarily based on their factor loadings and the proportion of variance explained. Due to the smaller dataset, it was considered optimal to utilise an Agglomerative Hierarchical Clustering technique to identify homogeneous sample groups based on sensory characteristics and/or physicochemical parameters. The optimal number of clusters was determined using the elbow method on the within–cluster inertia plot, specifically at the point where the rate of reduction shows a noticeable slowdown. Additionally, a linear regression analysis was performed between the set of sensory descriptors and several physicochemical parameters of the samples, believed to be directly related to the winemaking methods. Statistical tests were chosen based on Jackson [23] and Qannari & Schlich [29].

## 3. Results

### 3.1. Physicochemical Parameters

For all tastings, 0.75 L bottles purchased from the same source were used in groups of three for each wine sample and prepared for the tasting flight. All samples were analysed, and the measured values of the physicochemical parameters are listed in Table 3. The physicochemical parameters were measured in triplicate, and the results are presented as the mean and standard deviation. The values shown are consistent with OIV standards for determining physicochemical parameters. Regarding density, the OIV-MA-AS2-01B: R2009 method, Type IV, was used [30].

### 3.2. Data Concerning the Context of Tasting Sessions, Whether Using Opaque or Transparent Glasses

**Figure 2:**
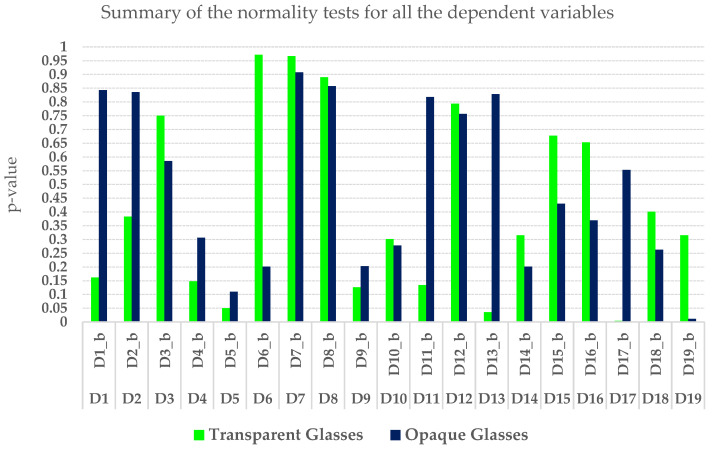
Shapiro–Wilk’s normality test of the dependent variables pertains to the tasting sessions, which are conducted either with opaque glasses to block visual cues or with transparent glassware.

As shown in Figure 2, for tasting sessions with suppressed visual stimuli, the dark-blue columns, all sensory descriptor values except for the ‘General Evaluation’ (D19_b), which has a ‘W’ of 0.924 but a ‘*p*-value’ less than 0.05, fall within the normal distribution range. This indicates a low likelihood of false-positive results or missing genuine differences. Furthermore, in Figure 2, for tasting sessions with transparent glasses, the green columns, descriptors such as green/fresh/citrus fruit, acidity/sourness, and unctuousness, had *p*-values below 0.05 for the Shapiro–Wilk W coefficient. This suggests a significant risk of false-positive results or failing to detect actual differences, which may lead us to dismiss these findings as unreliable.

In the opaque-glass tastings, six descriptors (maceration stage and its perceived intensity, red fruits, berries/forest fruits, Maillard-type notes, astringency, and other specific notes) showed highly significant effects (Pr > F < 0.0001), with R^2^ values ranging from 75.3% to 91.7%. In the transparent-glass tastings, five descriptors (maceration stage and its perceived intensity, red fruits, berries/forest fruits, spice notes, and astringency) were similarly significant (Pr > F < 0.0001), with R^2^ values ranging from 72.3% to 94.1%. Additional descriptors reached significance at Pr > F < 0.05 in both conditions—seven in the opaque-glass tastings (overall olfactory intensity, vegetal/herbal notes, green/fresh/citrus fruits, exotic/stone/tropical fruits, overripe fruits, spice notes, and bitterness) and four in the transparent-glass tastings (floral notes, Maillard-type notes, other specific notes, and bitterness). Across models, variance multiplier coefficients were mainly negative, a trend most pronounced among descriptors with the strongest significance (Pr > F < 0.0001).

**Figure 3:**
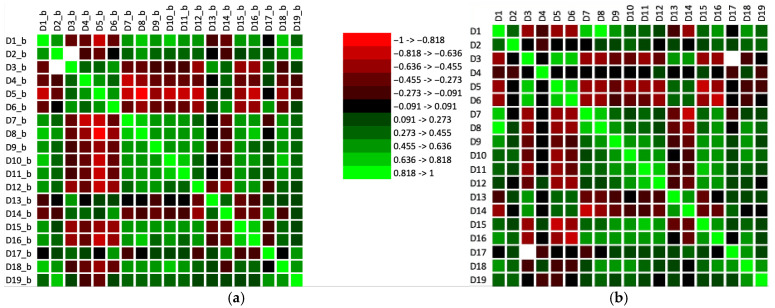
Image of the correlation matrix for sensory descriptors. (**a**) Tasting session with opaque glasses; (**b**) Tasting sessions with transparent glasses. D_x_b, x = 1–19_, represents statistical values for sensory descriptors (detailed in Table 2) recorded during tasting sessions with opaque glasses, while D_x, x = 1–19_, has the same meaning but for tasting sessions with transparent glasses.

The overall view of the correlation matrix of sensory descriptors related to tastings with opaque and transparent glasses, respectively, is shown in Figure 3.

**Figure 4:**
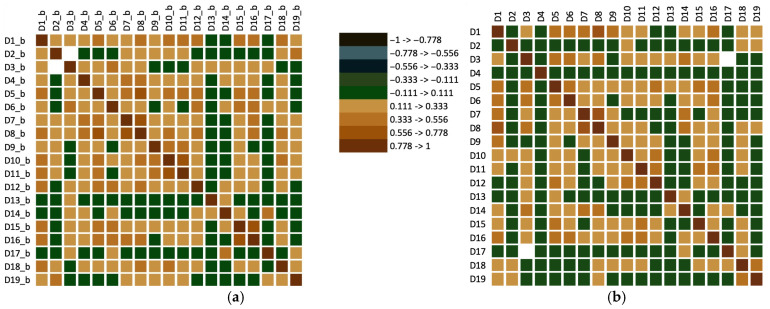
Image of the matrix of coefficients of determination (Kendall) for sensory descriptors related to tasting with opaque glasses. (**a**) Tasting session with opaque glasses; (**b**) Tasting sessions with transparent glasses. D_x_b, x = 1–19_, represents statistical values obtained for sensory descriptors (listed in Table 2) recorded during tasting sessions with opaque glasses, whereas D_x, x = 1–19_, has the same meaning but pertains to tasting sessions with transparent glasses.

Even with opaque glasses, the dependent variable ‘Existence of a maceration stage and perceived intensity of it’ shows the strongest positive correlation with descriptors such as Red fruits, Berries/forest fruits, Overripe fruits, Spice notes, Maillard–type notes, and Other specific notes in the case of red wines. This connection aids in characterising the wine, even when tasting blindly. If these notes are present, it is reasonable to infer that the winemaking process involved ‘red techniques’; if absent, it suggests a white wine winemaking method was used. According to subfigure ‘b’, the effect of transparent glasses on tasters’ bias becomes more apparent, as does the direct link between ‘Existence of a maceration stage and perceived intensity of it’ and descriptors like red fruits and berries/forest fruits. Furthermore, a similarity between the two tasting methods is evident in the dependent variables of the gustatory descriptors. The detailed relationship between pairs of descriptors is shown in Figure 4. Additionally, it is noteworthy that the descriptors ‘Red fruits, Berries/forest fruits, and Overripe fruits’ are considerably more significant factors for ‘Existence of a maceration stage and perceived intensity of it’ when tasting with transparent glasses, compared to opaque glasses.

**Figure 5:**
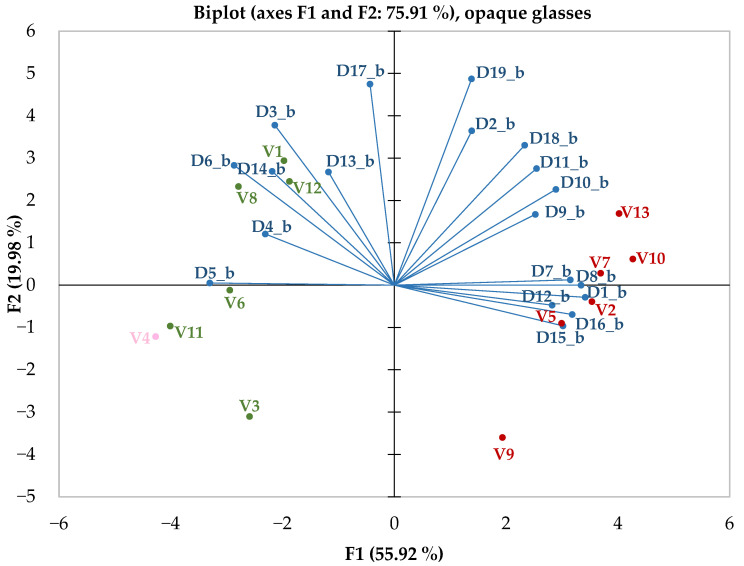
Principal Component Analysis during opaque glasses tasting sessions. D_x_b, x = 1–19_, represents statistical values obtained for sensory descriptors (detailed in Table 2) recorded throughout tasting sessions with opaque glasses. V_i, i = 1–13_, indicates the ID parameters of the samples according to the breakdown shown in Table 2. Red colour is used for red wines, green colour for white wines, pink colour for the sole rosé wine, and blue colour for dependent variables represented by sensory descriptors.

To enhance the separation of wines demonstrating winemaking techniques, a principal components analysis was conducted based on variables including sensory attributes outlined by the descriptors. The numerous sensory descriptors gathered during tastings, which were performed with opaque glasses, were condensed into two main explanatory factors, accounting for approximately 76% of the total variance, as shown in Figure 5.

The primary factor, accounting for about 56% of the data’s variability, emerges as a highly effective discriminator between red and white wines. Its structure is heavily influenced by sensory descriptors encountered when tasting red wines, such as red fruits, berries/forest fruits, spice notes, Maillard–type notes, other specific notes, bitterness, and astringency. The usefulness of this descriptive parameter is evidenced by the clear grouping of the two main wine categories, red and white. Since rosé wine was only available in a single sample, it cannot be included in the current comparison. The most suitable criteria for identifying red wines, based on the factor mentioned earlier, are the detection of odours or descriptors positively correlated with the main factor. Conversely, the absence of these indicators signifies that the wine is white. The second factor, which accounts for approximately 20% of the data’s variability, appears to be more relevant for identifying white wine through the alignment of acidity and floral notes.

**Figure 6:**
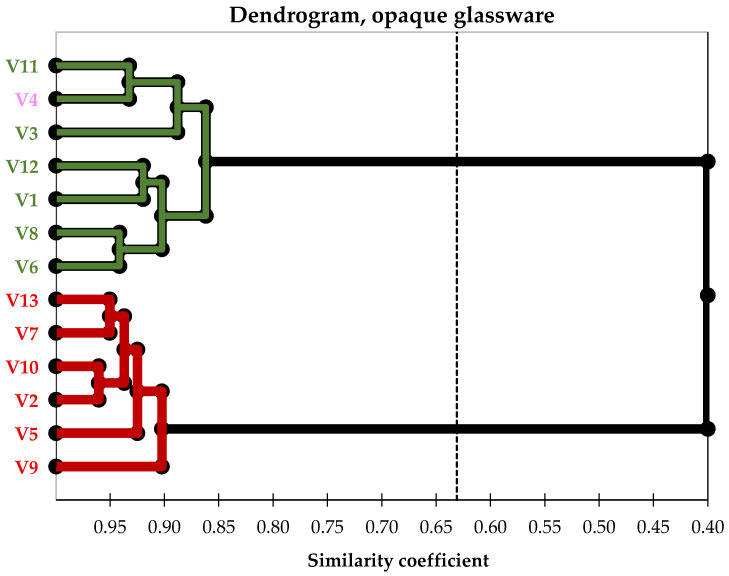
Dendrogram generated for opaque glasses tasting sessions. C1—cluster 1, green colour, mainly white wines but also the only rosé wine; C2—cluster 2, red colour, exclusively red wines; V_k, k = 1–13_, represent the ID of the wine sample used, with details provided in Table 1.

Based on the clustering structure revealed by the principal components analysis, as shown in Figure 5, the agglomerative hierarchical clustering method identifies the same pairs of relationships between samples. In Figure 6, the two main clusters are clearly visible. Optimal segmentation is determined using the ‘elbow’ rule, which finds the best number of clusters by identifying the point where within–cluster inertia decreases significantly.

**Figure 7:**
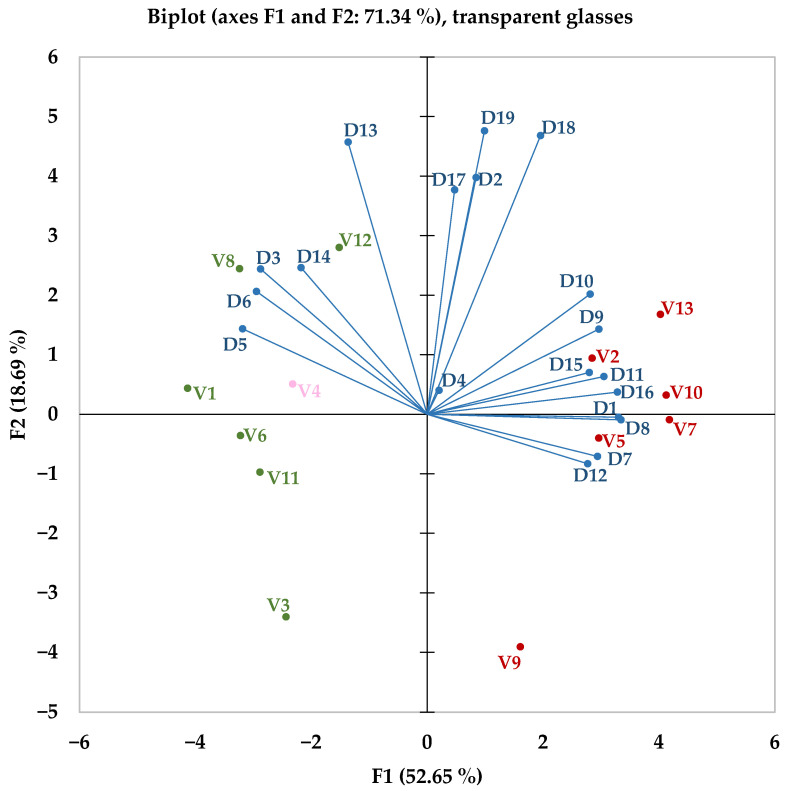
Principal Component Analysis of tasting sessions with transparent glassware. Red colour is used for red wines, green colour for white wines, pink colour for the sole rosé wine, and blue colour for dependent variables represented by sensory descriptors. D_x, x = 1–19_, represents statistical values obtained for sensory descriptors (detailed in Table 2).

To ensure consistency, the information gathered from tasting with transparent glasses was also organised. As illustrated graphically in Figure 7, the clustered pattern of the wine samples is clearly highlighted. The factor F1, which accounts for approximately 53% of the data variability, is shown on the horizontal axis, while the F2 factor, explaining around 19% of the variability, is displayed on the vertical axis.

The data from tasting with transparent glasses closely resemble those from tasting with opaque glasses. The main factor remains approximately at the same level of explained variability and is characterised by the same descriptors. These features reasonably indicate that opaque glasses have a lesser influence on tasters when differentiating between red and white wines. Additionally, for the second factor, the acidity descriptor follows the same trend, but somewhat strangely, the white wines appear to be more dispersed along the axis.

**Figure 8:**
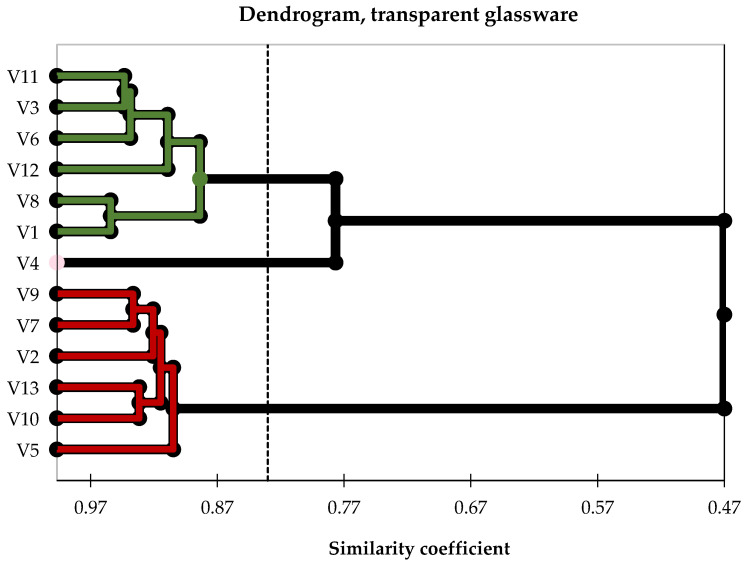
A dendrogram was created for the tasting sessions with transparent glasses. C1–cluster 1, green colour, contains only white wines; C2–cluster 2, red colour, includes exclusively red wines; C3–cluster 3, pink colour, consists solely of rosé wine; V_k_, where _k = 1–13_, represents the ID of each wine sample used, with details provided in Table 1.

Although visual stimuli from tasting in transparent glasses are presented, the visual cues already clearly reveal the grouping of samples based on the colour of the wines, as shown in Figure 8. The other perceived sensory descriptors grouped the samples into slightly different pairs, even though the ‘clusters’ identified by ‘elbow’ rules are similar to those associated with the tastings in opaque glasses.

**Figure 9:**
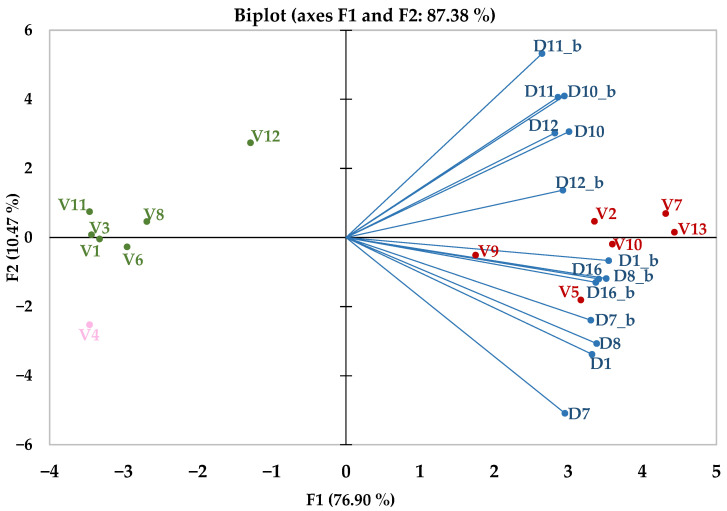
Principal Component Analysis based on overlapped tasting contexts, including opaque and transparent glasses. Red colour indicates red wines, green colour for white wines, pink colour for the sole rosé wine, and blue colour for dependent variables represented by sensory descriptors. D_x, x = 1–19_, represents statistical values obtained for sensory descriptors (detailed in Table 2).

For sensory descriptors that showed a statistically significant probability (<0.0001) of obtaining the F statistic value under the null hypothesis, a Principal Components Analysis model was utilised to verify, by comparing the statistical contexts associated with the two types of tastings—without and with visual stimuli—the biplot representations of the tasters’ notes. As illustrated in Figure 9, the tasters were not significantly misled by the absence of visual stimuli. The factor F1, which accounts for approximately 77% of the data variability, is represented by the horizontal axis, while the F2 factor, explaining 10% of the data variability, is depicted by the vertical axis.

**Figure 10:**
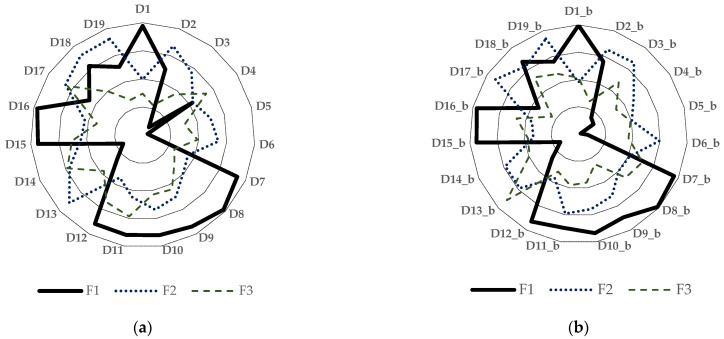
Principal Component Analysis specific to tasting contexts, including opaque and transparent glasses. (**a**) reflects the context of the tasting with opaque glassware; (**b**) reflects the context of the tasting with transparent glassware. D_x_b/x, x = 1–19_, denotes statistical values obtained for sensory descriptors (detailed in Table 2) during tasting with opaque glasses and transparent glassware.

These seven sensory descriptors appear to be sufficient for identifying red wines by their presence. Their absence mainly suggests a white wine in the glass. The clustering of the detected values near the main factor axis and their placement in the same quadrant for each descriptor, observed in both tasting conditions, whether in opaque or transparent glasses, emphasises that the lack of visual cues did not mislead the tasters. This limited influence of visual stimuli suppression on the differentiation by expert tasters of wines produced by the red vinification method compared to those made by the white vinification method is highly indicative in a typical type of statistical diagram used in the wine sector: the spider diagram. As shown in Figure 10, the first three factors, which account for over 80% of the difference between red and white wines, are heavily influenced by similar sensory descriptors, so much so that the shape of the descriptive zone remains remarkably consistent, regardless of whether the glass is opaque or transparent.

### 3.3. Data Related to the Context of the Winemaking Method

**Figure 11:**
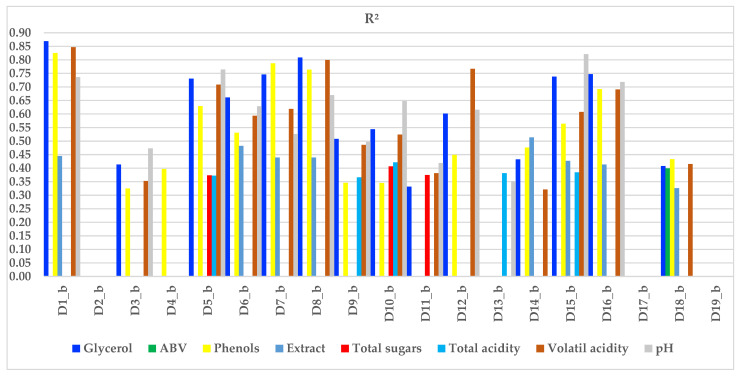
The R^2^ coefficient of determination for the significant *p*–value. The dependent variables, the sensory descriptors marked with D_x_b_, are displayed on the *x*-axis, with x = 1–19 corresponding to those listed in Table 2. The *y*-axis indicates the level of explanatory power of each independent physicochemical parameter’s influence on the dependent variables. Only when Pr > F is less than 0.05, and the closer to 1, the more relevant it is.

To ensure substantial variability among the analysed samples, wines produced using different vinification methods were selected. The difference between a ‘Blanc de noir’ and a red wine, both made from the same harvest of black grapes, is evident. The primary physicochemical factors influencing the sensory differences between these wines primarily result from the transfer of compounds from the grape skin to the wine during maceration via the must. Ultimately, the variability of sensory descriptors related to physicochemical parameters was confirmed using a linear regression model for each key parameter identified through instrumental analysis. To provide a comprehensive view of how the main physicochemical parameters influence the sensory descriptors highlighted in the sensory sheet, the graphical representation was limited to values where Pr > F is less than 0.05. Figure 11 illustrates the explanatory power of the independent physicochemical parameter on the dependent variable (sensory descriptors).

Glycerol, a polyhydric alcohol, shows high correlation values ranging from 0.864 to 0.932 in the correlation matrix with sensory descriptors typically associated with red wine traits, such as the presence of a maceration stage and perceived intensity of it, red fruits, berries/forest fruits, bitterness, and astringency. This aligns with the fact that the glycerol levels in wines, which originate from alcoholic fermentation, tend to be higher when the fermentation temperature matches the common red wine range of around 29 to 31 °C [31]. Phenols also exhibit strong correlations with these same descriptors, particularly in relation to the red wine winemaking method. Volatile acidity, a byproduct of fermentation, results from saturated aliphatic monocarboxylic acids [31]. Volatile acidity, mainly perceived in the sensorial profile due to acetic acid, is suppressed by the must’s low pH level and can be elevated by extended contact with berry grape skins. Typical wine pH values range from 2.8 to 3.8, with no direct link between total acidity and pH. In the study, wine pH, which increases with the neutralisation of tartaric acid, varies from 3.11 to 3.67. The highest values (3.56 to 3.67) were observed in red wines made from black grapes cultivated in hot regions. Seven of nineteen sensory descriptors commonly linked to red wines, such as presence of a maceration stage and perceived intensity of it, berries/forest fruits, overripe fruits, Maillard–type notes, other specific notes, bitterness, and astringency, show a high correlation coefficient with pH values. Lactic acid, a monocarboxylic acid, reduces perceived acidity in wine; the greater the amount of malic acid, the more notable this reduction. Only the red wines used in the research had a supraunitary lactic acid value.

**Figure 12:**
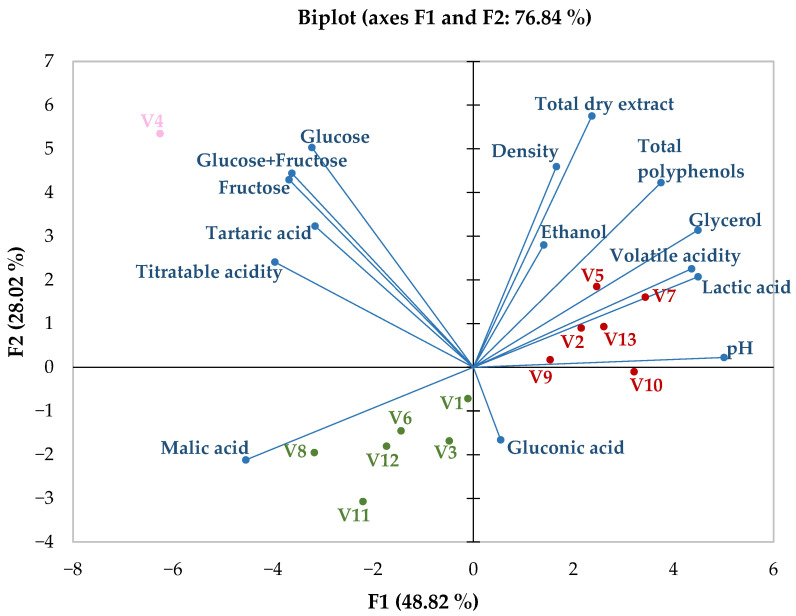
Principal component analysis based on physicochemical parameters. Red colour is used for red wines, green colour for white wines, pink colour for the sole rosé wine, and blue colour for dependent variables represented by physicochemical parameters.

Furthermore, Figure 12 presents an image displaying the distinctive clustering of the ‘Blanc de noirs’ and red wine samples used in this study, based on the identified physicochemical parameters. The F1 factor, which accounts for approximately 49% of the variation in the data, is plotted on the horizontal axis. Conversely, the F2 factor, accounting for roughly 28% of the variation, is shown on the vertical axis. Considering the temperature characteristics of the red winemaking process and the time the must spends in contact with the berry skin and stem, Figure 12 illustrates the relationship between this type of wine and its physicochemical composition, which ultimately influences the sensory profile.

In Figure 12, the isolated position of the coordinates related to wine sample V4 can be observed, which results from the high levels of total glucose and fructose, at 6.8 g/L, compared to the other samples, which had an average of 0.42 g/L with a standard deviation of 0.35 g/L. The rosé sample was not analysed further, as it was a single wine. Red wines are directly correlated with F1, explaining approximately 49% of the variability, while white wines are inversely associated with F1.

## 4. Discussion

### 4.1. Effects of Winemaking Method on Sensory Perception

This study aimed to determine whether a consistent pattern exists in the language and sensory knowledge used by experts when describing the sensory characteristics of different wine types made from the same grape varieties. Some interactions were clearly identified, while others remain intuitive. For example, glucose can increase volatility, while ethanol can decrease it [23]. The wines included in the research had residual sugar levels below 1, with an average of 0.42 and a standard deviation of 0.33; therefore, from this perspective, no significant variations were evident. Mannoproteins can suppress the volatility of flavour compounds such as β-ionone, ethyl hexanoate, and octanal, while promoting the release of others, including ethyl octanoate and ethyl decanoate [32]. Other examples related to the varied influences of wine’s compounds on volatility include the suppressive action of polyphenolics [33], glycerol [34], and polysaccharides [35].

Olfactory and gustatory sensory associations, including those from synesthetic aspects, can be enhanced or diminished depending on vinification methods or technological processes applied during grape processing. This variability results from different vinification techniques and procedures, which inherently involve a variety of compounds extracted from grape berry components or produced during maceration, alcoholic fermentation, malolactic conversion, and ultimately, maturation and/or ageing. The extraction of grape aroma compounds, found in both free and glycosidically bound forms within the mesocarp vacuoles and pericarp, mainly takes place through diffusion and leaching. The rate and extent of this extraction depend on factors such as the nature of the compound, its concentration, location within the grape berry, and the specific processing conditions used. Additionally, factors influencing extraction include temperature, duration, and intensity of maceration; the degree of clarification; and the solubility of the compound in the water–alcohol mixture. Beyond all these factors, the concentration gradient between grape solids and wine, along with the chemical equilibria and reactions during the fining process [35], can be encompassed under the term ‘winemaking method’. All these compounds extracted from the grape, which reflect the winemaking method, are measured using the Total dry extract parameter. The present research clearly differentiates between white and red wines, with the average level of total dry extract for whites being 20.03 g/L, while for red wines it was 43% higher. This difference was highlighted by the distinct clustering of red wines even when tasters used opaque glasses.

Brochet & Dubourdieu [36] argued that wine descriptions are inherently personalised and mainly meaningful to the taster, with experts demonstrating limited ability to identify wines solely based on descriptions provided by others. Although roughly 3% of the estimated ten thousand volatiles in food products contribute to the volatile stimulus space across different food and beverage categories, it has been found that as few as forty key odourants in a food item significantly influence its primary odour [37]. This observation also applies to alcoholic beverages. These approximately forty key odourants can be divided into two main groups. The first, known as the ‘aroma buffer’ [38], consists of twenty–seven components containing aroma molecules derived from alcoholic fermentation, such as isoamyl alcohol, isovaleric acid, and ethyl hexanoate [39]. These components predominantly exhibit sensory traits linked to fruity and floral qualities. The second group, labelled ‘aroma vectors’, includes molecules or groups of molecules that interfere with the ‘aroma buffer’ effect. They can be regarded as the fundamental ‘aroma units’ responsible for transmitting specific aroma nuances [39]. ‘Aroma vectors’ are olfactory stimuli that can be clearly identified and classified based on their distinctive odour profile [40]. The ‘aroma buffer’ is consistently present in both white and red wines, with only certain ‘vector molecules’ or their structural analogues able to influence this aromatic buffer [41]. Without these vector molecules, wines may display olfactory neutrality, making it difficult to distinguish between different grape cultivars. Since tasters can successfully distinguish between red and white wines even when using opaque glasses, it can be assumed that the aroma buffer, along with the aroma vector, provided by winemaking methods, is sufficient to produce an olfactory threshold low enough to be surpassed by personal limits.

The alignment of individual sensory perceptions can be understood by recognising recurring patterns, as human cognition naturally compares new stimuli with existing frameworks shaped by past experiences. These cognitive schemas serve as mechanisms to reduce uncertainty, enhance predictability, and foster a sense of control over subsequent interpretations. In practice, tasters tend to structure their interactions with wine samples more based on sensations than perceptions. Sensation, in contrast to perception, functions to detect stimuli, while perception assigns a meaning to them [42]. Sensation refers to the conscious or neurological response triggered by the stimulation of a sensory organ, nerve, or brain region. It encompasses the physiological process by which sensory organs (such as the eyes, ears, nose, tongue, and skin) respond to external stimuli, producing a perceived experience. Furthermore, sensation includes the mental processes (such as seeing, hearing, or smelling) that result from direct external stimulation of the sensory organs [42]. One of the key aspects of sensory parameter analysis is that the sense of smell accounts for 75–95% of the influence on flavours. This is further supported by the fact that around 75% of human emotions are triggered by odours linked to pleasure, well–being, emotion, and memory [42]. A finding from the present study, as shown in Figure 9 and Figure 10, suggests that the sense of smell can override the absence of visual cues, particularly for experienced tasters.

Even when tastings were conducted using either opaque or transparent glasses, the tasters’ notes clustered the red wines based on descriptors related to the presence and perceived intensity of a maceration stage, red fruits, berries/forest fruits, along with other specific notes, bitterness, and astringency. Furthermore, the values associated with these descriptors correlated with those linked to overripe fruits and spicy notes. All statistical data for these descriptors indicate a direct relationship with wines produced using red winemaking techniques. Given the blind tasting conditions where participants were unaware of the samples’ origins, and considering Lesschaeve’s findings that experts can effectively utilise their cognitive faculties, though when they know the wines beforehand, they can more precisely articulate sensory perceptions, even when specific components are not detected [43], the grouped values of the tasters’ notes suggest that sensory features are connected to winemaking methods.

The sensory language used by experts in wine descriptions is organised within a framework defined by typological classifications. A wine evaluator does not conduct a separate analysis of individual sensory traits; instead, they make a comparative assessment of the overall cognitive associations evoked by the sample. Terminological conventions used by connoisseurs maintain a consistent connection with the wine’s colour properties; the complex organoleptic profile is then expressed through descriptors typically associated with objects of a similar nature or shades [44]. The sensory detection of green fruit, citrus notes, and stone fruit components (qualities strongly associated with yellow hues) showed statistically significant improvement in the evaluation of white wines. In contrast, the perception of red fruit, anthocyanin–rich fruit, and woody characteristics (factors linked with violet and ruby hues) exhibited notable enhancement in the organoleptic assessment of red wine samples [36].

In the white wine group, the main separation criterion identified was an inverse relationship with the F1 factor, indicating the influence of physicochemical parameters and a strong correlation with volatile acidity, lactic acid, and pH levels. Sample V4 shows a significant deviation from both wine colour groups, driven by the F2 factor, which has strong correlations with parameters related to residual sugars. The V4 sample was not further analysed because it was solely rosé wine.

Based on the structured olfactory framework and integrative cognitive associations in the case of white wines, the identification of the olfactory characteristics of monoterpenes, which have a strong aroma and low sensory thresholds [45], can trigger the entire sensory evaluation process.

Conventional classifications recognise three main categories of white wines based on the level of Muscat aroma concentration [46], as shown in Table 4.

### 4.2. The Influence of Tasting Context on Sensory Perception

In addition to differences caused by the winemaking method, the effects of visual stimulus suppression were also examined. The statistical data from tasters’ impressions recorded on the sensory sheets indicate that mainly the absence of certain features influences the sensory profile used to assess the white wine samples. The descriptors with significant statistical values, which explain a large part of the variation, are those directly related to wines produced through the red vinification method. As clearly shown in Figure 9, the values associated with the tasting sessions using opaque (D_x_b_) and transparent glasses (D_x_), respectively, for each sensory descriptor—including the existence of a maceration stage and its perceived intensity, red fruits, berries/forest fruits, spicy notes, Maillard notes, other specific notes, and astringency—are located in the same quadrant. The grouping pattern of red wines, in direct correlation with the respective descriptors, and white wines, in inverse correlation with the same descriptors, was also maintained.

Across primates in general, and humans in particular, visual processing serves as the primary sensory modality, guiding overall perceptual integration. In gustatory perception, this visual dominance is especially significant, as visual input—particularly chromatic information—substantially influences our perceptual interpretation and identification of edible and drinkable substances [47]. Consistent with the hypothesis that oenological knowledge structures may be organised according to chromatic dimensions, a white wine artificially tinted red elicited significantly more red wine olfactory descriptors in participant evaluations than the same wine in its natural, uncoloured state [48].

The present research, based on statistical data, reveals a notable positive correlation between sensory descriptors characteristic of the maceration stage of black grape varieties, as evaluated solely through olfactory assessment. This includes descriptors such as berries/forest fruits as well as astringency, even when visual cues were excluded from the sensory evaluation protocol. Both descriptors show a high direct correlation with the group of red wines in Principal Component Analysis (Figure 4). Furthermore, a strong inverse correlation was identified between the values associated with vegetable notes and green/fresh/citrus fruits, and those associated with red fruits and berries/forest fruits.

Some studies suggest that recognising wine colours as either white or red by smell alone, with visual cues altered, generally performs poorly. The descriptors used to characterise a wine’s aroma are primarily influenced by its colour, with colour associations often embedded within descriptive narratives, even if not explicitly mentioned. Empirical evidence indicates that when the colour of white wine is artificially altered to resemble that of red wine, it prompts olfactory perceptions typically associated with red wine. These findings imply that a wine’s colour conveys significant sensory information, which can affect individuals’ objective assessment of its taste qualities. Moreover, this perceptual bias is more pronounced when visual access to the wine’s colour is available, compared to situations where such visual information is absent [48]. Conversely, other studies have demonstrated that tasters, regardless of their oenological expertise, have a statistically significant ability to distinguish between red and white wines using only olfactory cues, while failing to reliably identify rosé varieties under the same sensory restrictions [2].

Summarising the results of this study, it is reasonable to suggest that, in the absence of wine colour indicators, tasters generally demonstrate a fair ability to identify the group to which the sample belongs. For red wines, this discrimination is based on direct correlations with specific descriptors, especially those associated with glycerol, phenols, volatile acidity, and pH. Conversely, white wine separation appears to be achieved through inverse correlations with these same physicochemical parameters, where lower values seem to assist in the identification of white wines. Previous research suggests that the sequence of wine presentation, with transparent glasses followed by opaque ones, may have influenced judges’ assessments, leading to higher scores for aroma intensity, possibly due to the influence of expectations. Furthermore, when wines were served in transparent glasses, descriptors containing terms that inherently imply colour were rated at higher intensities [45]. The present study was conducted solely with the variant in which the visual stimuli were suppressed and did not involve the variant of altering the chromatic cue. Additionally, only tasters with regulated wine appraisal were involved.

## 5. Conclusions

This study found that a panel of experts could accurately distinguish between white and red wine samples produced from the same grape variety and with visual cues intentionally suppressed with statistically significant accuracy. For red wines, it was observed that sensory perceptions influenced by the prominent presence of glycerol, phenols, volatile acidity, and a higher pH level, characteristic of the winemaking process, are key factors in correct identification. In the case of white wines, a negative correlation among descriptors suggested that the same physicochemical parameters remain important, albeit through an indirect mechanism characterised by their non-detection. Key sensory attributes—such as the presence and intensity of maceration, berry and forest-fruit notes, astringency, and red-fruit character—were consistently recognized by tasters during blind evaluations as distinguishing red from white wines, independent of their sensory background. However, recognising these parameters implies a high likelihood that a sample is from red wine vinification, whereas their absence suggests it is from white wine vinification. Eliminating visual cues does not significantly alter the fundamental features of either type of wine to the point where tasters cannot distinguish between them. Therefore, it can be concluded that if a wine is selected based on its colour and its sensory profile does not meet expectations, the chances of consuming it again are reduced. To overcome the limitations related to assumptions about chromatically altered tasting samples, the inclusion of rosé wines, and the reliance on a small panel composed exclusively of experts, a follow-up experiment will be conducted. The study’s findings can be applied in the hospitality sector, particularly by specialised wine bars, to develop collections and recommend wines to consumers based on their individual sensory preferences, thereby ensuring satisfaction. Moreover, the results could assist producers in defining product specifications and aid authorities in establishing regulations to provide a more precise description of the organoleptic characteristics of wines with Protected Designation of Origin. Last but not least, professional taster organisations can establish procedures to refine the panel used in product certification by arranging tastings with both opaque and transparent glasses in advance to evaluate tasters’ acuity.

## Figures and Tables

**Table 1:** Breakdown of wine samples from the ‘line–up’ used in the study.

ID	Vintage	Grape Variety	Vinified	Winery	Region
V1	2021	Bobal	Blanc de noir	Vicente Gandia	ES–Utiel Requena
V2	2020	Bobal	Red	Vicente Gandia	ES–Utiel Requena
V3	2021	Cabernet Sauvignon	Blanc de noir	Rojevas Agroinvest	RO–Târgu Bujor
V4	2022	Cabernet Sauvignon	Rosé	Rojevas Agroinvest	RO–Târgu Bujor
V5	2021	Cabernet Sauvignon	Red	Rojevas Agroinvest	RO–Târgu Bujor
V6	2021	Cabernet Sauvignon	Blanc de noir	Domaine Vinarte	RO–Sâmburești
V7	2017	Cabernet Sauvignon	Red	Domaine Vinarte	RO–Sâmburești
V8	2019	Tinta Negra	Blanc de noir	Diana Silva Wines	PT–Madeira
V9	2019	Tinta Negra	Red	Diana Silva Wines	PT–Madeira
V10	2019	Tinta Roriz	Red	Quinta do Vallado	PT–Douro
V11	2021	Tinta Roriz	Blanc de noir	Quinta Nova	PT–Alto Douro
V12	2021	Touriga Franca (95%)	Blanc de noir	Ravasqueira	PT–Alentejo
V13	2020	Touriga Franca (95%)	Red	Ravasqueira	PT–Alentejo

**Table 2:** Monitored organoleptic characteristics of wine samples.

Conventional Ordinal Number Allocated in the Representation of Statistical Values *	Descriptor Title	Description of the Specific Features/Notes Appraised by Descriptor	References
1	Existence of a maceration stage and perceived intensity of it	Presumed exclusively based on orthonasal olfactory stimuli in tasting sessions with opaque glasses.	–
2	Overall olfactory intensity	Exclusively based on orthonasal olfactory stimuli.	–
3	Floral notes	Blossom notes, consisting in elderflower, honeysuckle, jasmine, rose, violet, acacia, chamomile, linden, honey, geranium odors/aromas	[25]
4	Vegetal/herbal notes	Eucalyptus, mint, fennel, dill, dried herbs thyme, oregano, lavender odors/aromas	[25]
5	‘Green’/fresh/citrus fruits	Apple, pear, gooseberry, grape–fruit, orange, grape odors/aromas	[25]
6	Exotic fruits/stone fruits/tropical fruits	Peach, apricot, nectarine, plum banana, melon, watermelon, passion fruit, pineapple odors/aromas	[25]
7	Red fruits	Redcurrant, cranberry, raspberry, strawberry, red cherry, red plum, pomegranate odors/aromas	[25]
8	Berries/forest fruits	Blackcurrant, Blackberry, blueberry, Black cherry, Black plum, sour–cherry odors/aromas	[25]
9	Overripe fruits	Figs, dried plums, raisins, prune, jam odors/aromas	[25]
10	Spice notes	Cinnamon, pepper, cloves, saffron, vanilla, coconut, liquorice, cedar, nutmeg, anise odors/aromas	[25]
11	Maillard–type notes	Roasted hazelnut/walnut, almond, coal smoke, cocoa, coffee, caramel, chocolate, toast, resins, tobacco odors/aromas.	–
12	Other specific notes	Leather, tanned leather, mushrooms, wet stone, flint, red earth, eucalyptus odors/aromas	[25]
13	Acidity/sourness	Fresh or sour taste produced by the natural organic acids, one of the primary tastes sensed by tastebuds on the tongue	[26]
14	Sweetness	One of the primary tastes involved in tasting, mainly because of the amount of residual sugar they contain	[26]
15	Bitterness	Among primary tastes which can be detected via taste buds mainly on the tongue, often confused with the quite different tactile sensation caused by astringency	[26]
16	Astringency	A complex tactile response resulting from shrinking, drawing, or puckering of the tissues of the mouth, based in principle by binding between tannins with proteins	[26]
17	Unctuousness	More a perceptual descriptor to describe the physical property of viscosity understood as the quality sensed by the human palate in the form of resistance as the solution is rinsed around the mouth	[26]
18	Finish/post–taste persistence	Somehow derided tasting term to appraise the persistency of flavour and the impact of the wines on the palate, supposed to be direct proportional with some colloids	[26]
19	Overall evaluation	Based on summarizing all previous personal sensory judgments	–

* In related statistical analyses and interpretations, each ordinal number assigned to a descriptor title is preceded by the letter ‘D’ to indicate that the value reflects the aggregated statistical parameters of the entire tasters’ panel for that specific descriptor perceived during tasting sessions with transparent glasses. Similarly, the same concept applies to values summarising the statistical parameters of the whole tasters’ panel in tasting sessions with opaque glasses, which is denoted by ‘D_b’ placed before the ordinal number assigned to each descriptor title.

**Table 3:** Physicochemical parameters.

Parameter	V1	V2	V3	V4	V5	V6	V7
**Ethanol (% vol)**	12.24 ± 0.00	14.42 ± 0.01	12.08 ± 0.00	13.84 ± 0.01	11.36 ± 0.00	12.8 ± 0.00	14.32 ± 0.00
**Glucose + Fructose (g/L)**	0.90 ± 0.02	0.10 ± 0.01	0.80 ± 0.03	6.80 ± 0.02	n/d ± 0.00	0.90 ± 0.00	0.50 ± 0.02
Titratable acidity (g/L *)	4.73 ± 0.00	4.64 ± 0.00	4.57 ± 0.00	6.85 ± 0.01	5.5 ± 0.01	4.79 ± 0.00	4.69 ± 0.00
Volatile acidity (g/L **)	0.46 ± 0.01	0.69 ± 0.01	0.38 ± 0.00	0.33 ± 0.00	0.78 ± 0.01	0.37 ± 0.01	1.13 ± 0.01
**Malic acid (g/L)**	1.09 ± 0.01	n/d ± 0.00	1.33 ± 0.02	1.78 ± 0.02	n/d ± 0.00	0.68 ± 0.01	n/d ± 0.01
**Tartaric acid (g/L)**	1.11 ± 0.00	1.58 ± 0.01	0.99 ± 0.00	2.62 ± 0.02	1.81 ± 0.00	1.69 ± 0.00	1.13 ± 0.00
**Lactic acid (g/L)**	0.63 ± 0.00	1.22 ± 0.01	0.65 ± 0.00	n/d ± 0.00	1.93 ± 0.00	0.26 ± 0.00	1.53 ± 0.01
**pH**	3.40 ± 0.01	3.56 ± 0.01	3.47 ± 0.01	3.11 ± 0.00	3.56 ± 0.01	3.34 ± 0.02	3.67 ± 0.01
**Density (g/mL)**	0.9915 ± 0.01	0.9910 ± 0.00	0.9913 ± 0.00	0.9922 ± 0.01	0.9950 ± 0.01	0.9887 ± 0.00	0.9919 ± 0.01
**Total dry extract (g/L)**	24.50 ± 0.01	29.60 ± 0.02	23.40 ± 0.00	30.80 ± 0.00	30.70 ± 0.00	18.90 ± 0.01	31.60 ± 0.01
**Glycerol (g/L)**	8.00 ± 0.00	9.30 ± 0.00	7.00 ± 0.01	6.70 ± 0.00	9.30 ± 0.02	6.60 ± 0.01	9.90 ± 0.01
**Polyphenols–total (mg/L)**	1.28 ± 0.00	2.08 ± 0.00	0.49 ± 0.00	1.22 ± 0.00	2.64 ± 0.00	0.87 ± 0.00	2.27 ± 0.00
**Parameter**	**V8**	**V9**	**V10**	**V11**	**V12**	**V13**	
**Ethanol (% vol)**	11.66 ± 0.00	11.44 ± 0.01	14.43 ± 0.01	12.55 ± 0.00	12.43 ± 0.01	14.58 ± 0.00	
**Glucose + Fructose (g/L)**	0.30 ± 0.02	n/d ± 0.00	n/d ± 0.00	0.40 ± 0.01	0.70 ± 0.02	0.40 ± 0.01	
Titratable acidity (g/L *)	6.37 ± 0.00	5.09 ± 0.00	4.19 ± 0.00	5.33 ± 0.01	5.13 ± 0.01	4.66 ± 0.00	
Volatile acidity (g/L **)	0.43 ± 0.01	0.74 ± 0.01	0.77 ± 0.01	0.41 ± 0.00	0.38 ± 0.01	0.88 ± 0.00	
**Malic acid (g/L)**	2.11 ± 0.02	n/d ± 0.00	0.15 ± 0.00	1.95 ± 0.01	1.22 ± 0.01	n/d ± 0.00	
**Tartaric acid (g/L)**	2.11 ± 0.01	1.77 ± 0.01	0.51 ± 0.00	1.12 ± 0.00	1.49 ± 0.01	1.30 ± 0.00	
**Lactic acid (g/L)**	n/d ± 0.00	2.08 ± 0.01	1.55 ± 0.00	0.05 ± 0.00	0.22 ± 0.00	1.45 ± 0.00	
**pH**	3.20 ± 0.01	3.47 ± 0.01	3.66 ± 0.00	3.35 ± 0.01	3.33 ± 0.01	3.62 ± 0.02	
**Density (g/mL)**	0.9895 ± 0.02	0.9920 ± 0.01	0.9902 ± 0.00	0.9883 ± 0.00	0.9891 ± 0.01	0.9905 ± 0.01	
**Total dry extract (g/L)**	17.50 ± 0.01	23.30 ± 0.01	27.40 ± 0.02	17.10 ± 0.00	18.80 ± 0.01	28.80 ± 0.01	
**Glycerol (g/L)**	5.70 ± 0.00	8.30 ± 0.00	9.60 ± 0.00	5.70 ± 0.01	6.30 ± 0.00	9.70 ± 0.01	
**Polyphenols–total (mg/L)**	0.64 ± 0.00	1.80 ± 0.00	2.00 ± 0.00	n/d ± 0.00	0.75 ± 0.00	1.90 ± 0.00	

The results are presented as mean and standard deviation. * indicates tartaric acid equivalents to pH 7.0; ** indicates values expressed in acetic acid; n/d means non-detectable value. V_i, i = 1–13_, represents the ID parameters of the samples according to the breakdown shown in Table 2.

**Table 4:** Classification of some white varieties based on monoterpene contents.

Muscat Varieties > 6 mg/L	Non–Muscat, Aromatic Varieties 1–4 mg/L	Neutral Varieties < 1 mg/L
Canada Muscat	Traminer	Bacchus
Gewürztraminer	Huxelrebe	Chardonnay
Muscat of Alexandria	Kerner	Chasselas
Muscat blanc à petits grains	Morio–Muskat	Chenin blanc
Moscato Bianco	Müller–Thurgau	Clairette
Muscat Ottonel	Riesling	Nobling
Moscato Italiano	Scheurebe	Rkatsiteli
	Siegerrebe	Sauvignon blanc
	Sylvaner	Sémillon
	Würzer	Sultana
	Italian Riesling	Trebbiano
		Verdelho
		Viognier
		Vidal blanc

## Data Availability

The original contributions presented in this study are included in the article. Further inquiries can be directed to the corresponding author.
